# Undertriage of Trauma-Related Deaths in U.S. Emergency Departments

**DOI:** 10.5811/westjem.2016.2.29327

**Published:** 2016-05-02

**Authors:** Jenelle A. Holst, Sarah M. Perman, Roberta Capp, Jason S. Haukoos, Adit A. Ginde

**Affiliations:** * University of Colorado School of Medicine, Department of Emergency Medicine, Aurora, Colorado; † Denver Health Medical Center, Department of Emergency Medicine, Denver, Colorado

## Abstract

**Introduction:**

Accurate field triage of critically injured patients to trauma centers is vital for improving survival. We sought to estimate the national degree of undertriage of trauma patients who die in emergency departments (EDs) by evaluating the frequency and characteristics associated with triage to non-trauma centers.

**Methods:**

This was a retrospective cross-sectional analysis of adult ED trauma deaths in the 2010 National Emergency Department Sample (NEDS). The primary outcome was appropriate triage to a trauma center (Level I, II or III) or undertriage to a non-trauma center. We subsequently focused on urban areas given improved access to trauma centers. We evaluated the associations of patient demographics, hospital region and mechanism of injury with triage to a trauma versus non-trauma center using multivariable logistic regression.

**Results:**

We analyzed 3,971 included visits, representing 18,464 adult ED trauma-related deaths nationally. Of all trauma deaths, nearly half (44.5%, 95% CI [43.0–46.0]) of patients were triaged to non-trauma centers. In a subgroup analysis, over a third of urban ED visits (35.6%, 95% CI [34.1–37.1]) and most rural ED visits (86.4%, 95% CI [81.5–90.1]) were triaged to non-trauma centers. In urban EDs, female patients were less likely to be triaged to trauma centers versus non-trauma centers (adjusted odds ratio [OR] 0.83, 95% CI [0.70–0.99]). Highest median household income zip codes (≥$67,000) were less likely to be triaged to trauma centers than lowest median income ($1–40,999) (OR 0.54, 95% CI [0.43–0.69]). Compared to motor vehicle trauma, firearm trauma had similar odds of being triaged to a trauma center (OR 0.90, 95% CI [0.71–1.14]); however, falls were less likely to be triaged to a trauma center (OR 0.50, 95 %CI [0.38–0.66]).

**Conclusion:**

We found that nearly half of all trauma patients nationally and one-third of urban trauma patients, who died in the ED, were triaged to non-trauma centers, and thus undertriaged. Sex and other demographic disparities associated with this triage decision represent targeted opportunities to improve our trauma systems and reduce undertriage.

## INTRODUCTION

Regionalized trauma systems have been developed to improve outcomes after injury by preferentially triaging injured patients to designated trauma centers.^1^ Survival of trauma patients is higher at trauma centers, due to immediate and ongoing access to highly skilled clinicians and resources.^2^–^4^ Prehospital emergency medical services (EMS) trauma triage protocols have been developed to aid in identifying and triaging the most critically ill patients.^5^–^13^ These protocols are used to minimize both under- and overtriage of patients, in an attempt to match the acuity of the patient with the appropriate level of hospital care.^10^ The most severely injured patients should be transported to major trauma centers (Level I or II), if available at a reasonable distance, whereas patients with minor injuries may be taken to lower level trauma centers (Level III) or non-trauma-designated hospitals.

Inaccurate triage that results in a patient requiring a higher level of care not being transported to a trauma center is termed undertriage,^13^ and can lead to avoidable morbidity and mortality. If a patient with minor trauma is taken to a trauma center this is considered overtriage and can lead to unnecessary cost and burden on the limited number of trauma centers, as cost of maintaining a trauma center is considerable.^14^ Injured patients who ultimately die in the emergency department (ED) are the most critically ill subset of trauma patients and need to be appropriately triaged to a trauma center where they have the best chance of survival. Previous studies have assessed undertriage of trauma patients with varying degrees of injury severity, but have not individually assessed the most severely injured subset of patients who die in the ED.^15^–^17^

Level I and II trauma centers are most often centered in urban areas,^18^,^19^ while rural areas frequently have lower level or no nearby trauma center. With readily available access to tertiary care trauma centers, urban areas should have very low rates of undertriage if field triage criteria are used accurately and appropriately. When assessing undertriage, previous studies did not differentiate between urban and rural areas.^15^–^17^ This geographic distinction when assessing undertriage is important given the anticipated differences in trauma center availability in urban versus rural areas.

Triage of injured patients is one of the most important components of an effective regionalized trauma system, yet little is currently known about national rates of undertriage of severely injured patients who die in the ED. We hypothesized that since ED death is a marker of the most critically injured trauma patient, undertriage of this patient population would be rare, especially in urban areas with readily accessible trauma centers. However, rural areas, with their inherent limited access to trauma centers, were hypothesized to have higher rates of undertriage. Additionally, we sought to identify patient and hospital characteristics associated with undertriage and determine targeted opportunities to improve EMS triage decisions.

## METHODS

### Study Design and Population

This study was a retrospective, cross-sectional analysis of the 2010 National Emergency Department Sample (NEDS), Healthcare Cost and Utilization Project, Agency for Healthcare Research and Quality.^20^ The NEDS is the largest all-payer ED database in the United States. The NEDS provides patient-level data on a 20% stratified sample of ED visits from 969 hospitals in 29 states, of which 164 (17%) were designated trauma centers. Hospitals are selected using a stratified probability sample based on hospital characteristics to provide weighted national estimates of ED visits, which were approximately 129 million in 2010. Analysis of this publically available dataset was approved by the institutional review board.

We included all adult, age ≥18 years, trauma-related ED visit patients. Trauma-related ED visits were defined by the injury variable available in the NEDS, which uses injury-related International Classification of Diseases, 9^th^ edition, (ICD-9) codes in any diagnosis field as previously defined.^21^ We then restricted our cohort to patients whose ED visit resulted in death during the index ED visit (prior to hospital admission or transfer). The primary outcome was trauma center designation of the hospital (i.e., trauma center level I, II or III versus non-trauma center). We defined undertriage as a patient visit with a traumatic injury ending in death in the ED of a non-trauma center. NEDS obtains trauma center status from the Trauma Information Exchange Program database,^20^ which includes state designation or American College of Surgeons verification.

After initial overall descriptive analyses of deaths in EDs located in metropolitan (urban) and rural hospitals combined, we further restricted our cohort to ED deaths in urban hospitals to focus on a population with improved access to trauma centers. Metropolitan areas were defined in NEDS based on the county-based Urban Influence Codes (UIC)^20^: ≥50,000 people (metropolitan), non-metropolitan regions with <50,000 and >10,000 people (micropolitan locations), and rural locations contain <10,000 people (micropolitan and rural categories combined for this analysis). Patient socio-demographic characteristics included age, sex, median household income based on the patient’s zip code, and primary insurance/payer. Clinical characteristics included whether the ED visit occurred during a weekday or weekend day, month, and mechanism of injury, as defined by ICD-9 External-Cause-of-Injury-Codes.^22^ Additionally, we examined hospital characteristics including census region (Northeast, Midwest, South and West) ownership (public, private and combined, which were hospitals in strata too small to stratify based on control), teaching status, annual ED volume, and safety net status. Safety net status was defined as an ED with >30% ED visits with Medicaid, or self-pay/no charge (uninsured), or >40% ED visits combined Medicaid or uninsured.^23^

### Statistical Analysis

We used descriptive statistics to summarize the data. Among urban hospitals, we used multivariable logistic regression to estimate the associations between socio-demographic and clinical characteristics of ED trauma deaths with triage to trauma versus non-trauma centers. Characteristics were removed from the multivariate model if they were collinear with hospital trauma or urban status (i.e., teaching hospital and safety net status). Because there was <5% missing for each variable of interest, missing observations were dropped rather than imputed (final multivariable model with <10% missing data). We used survey commands to account for the complex survey design and provide national estimates, per NEDS guidelines. Analyses were conducted in SAS 9.3 (SAS Inc, Cary, NC) and Stata 12.1 (Stata Corp, LP, College Station, TX).

## RESULTS

**Figure 1:**
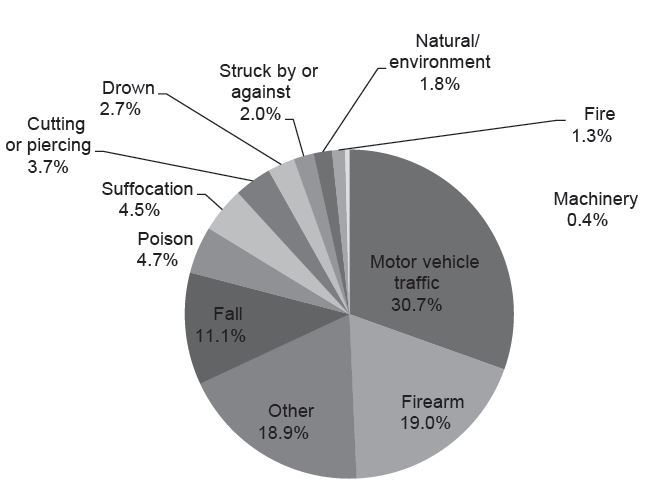
Mechanism of injury of US emergency department trauma-related deaths.

The 2010 NEDS contained 42,614 observations of adult ED deaths, of which 3,971 (9.3%) were trauma-related, representing 18,464 ED trauma deaths nationally. Patient, visit and hospital characteristics of these deaths are presented in Table 1. The largest demographic groups among trauma deaths were young (age 18–34 years), male, low median household income, and self-pay. The mechanisms of injury for ED deaths are displayed in Figure 1. The four most common mechanisms were motor vehicle trauma (MVT), including occupant of or person struck by an automobile or motorcycle (30.7%, 95% CI [29.2–32.2]), injury by firearm (19.0%, 95% CI [17.8–20.3]), other (18.9%, 95% CI [17.7–20.2]), and falls (11.1%, 95% CI [10.1–12.1]). Of all combined rural and urban U.S. trauma deaths, nearly half (44.5%, 95% CI [43.0–46.0]) of patients were triaged to non-trauma centers, and thus undertriaged.

**Figure 2:**
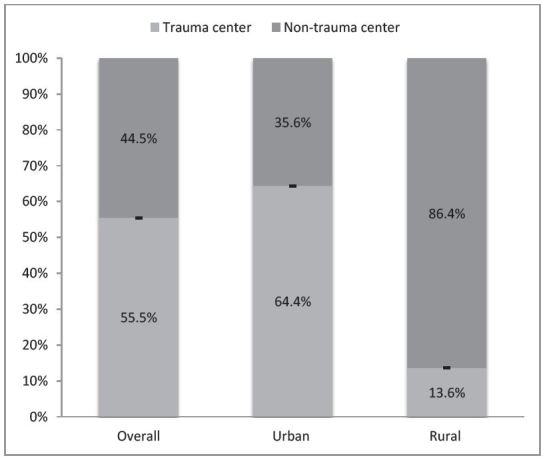
Trauma deaths triaged to trauma vs. non-trauma centers in urban, rural and overall emergency departments.

Figure 2 displays trauma vs. non-trauma center status of ED deaths in urban and rural locations. For patients taken to EDs in urban areas, most patients were triaged to trauma centers; however, still over a third (35.6%, 95% CI [34.1–37.1]) were triaged to non-trauma centers, and thus undertriaged. Most ED trauma deaths were triaged to non-trauma centers in rural areas (86.4%, 95% CI [81.5–90.1]).

Next our analysis focused on urban areas to further explore the characteristics associated with undertriage of ED trauma deaths triaged to non-trauma centers (descriptive results in Table 2). The multivariable logistic regression results for characteristics associated with triage to an urban trauma vs. urban non-trauma center are shown in Table 3. Female patients were less likely to be triaged to trauma centers versus non-trauma centers (adjusted odds ratio [OR] 0.83, 95% CI [0.70–0.99]). Highest median household income zip codes were less likely to be triaged to trauma centers than lowest median income (OR 0.54, 95% CI [0.43–0.69]). Compared to MVT, firearm trauma had similar odds of being triaged to a trauma center (OR 0.90, 95% CI [0.71–1.14]); however, falls were less likely to be triaged to a trauma center (OR 0.50, 95% CI [0.38–0.66]).

## DISCUSSION

In this study we found that nationwide, nearly half (44.5%) of trauma patients who died in the ED died in a non-trauma center. This is the most concerning form of undertriage because this subset of trauma patients who die in the ED are the most gravely injured and triage to a trauma center is crucial for improving their chances of survival. To our knowledge this is the first national study focusing solely on evaluating the destination and outcomes of the most critically ill trauma patients, those who die in the ED. Based on our results, the extent of undertriage of U.S. trauma patients who ultimately die in the ED is remarkably high. Previous studies estimated undertriage ranging from 34% to 69%; however, they studied less severely injured patients,^15^,^16^ or grouped ED deaths in non-trauma centers with other forms of undertriage such as patients treated and released from the ED of a non-trauma center or admitted to a non-trauma center.^17^ Identifying these critically injured patients in the prehospital setting and finding ways to ensure triage to a trauma center will potentially be the most impactful way to reduce undertriage and prevent mortality.

Our results also identified a large burden of undertriage in urban areas, where over a third (35.6%) of trauma patients who died in the ED died in a non-trauma center. This is the first national estimate of urban trauma undertriage as previous studies did not differentiate between urban and rural.^15^–^17^ This differentiation is important because accessibility to trauma centers is very different in urban versus rural areas. Trauma systems should be the most advanced in the urban setting given the closer proximity that Americans living in cities have to trauma centers. One study indicated that 84.1% of Americans have access to level I or II trauma centers within one-hour transport time, and these people live mostly in urban areas.^18^,^19^ This leaves 46.7 million living in mostly rural areas without trauma center access within an hour. Accordingly, we anticipated the observed difference in undertriage between rural and urban ED trauma deaths in our study. However, the large amount of trauma deaths in urban areas that were undertriaged to non-trauma centers was higher than anticipated.

Prehospital EMS trauma triage protocols have been developed to aid in identifying the most critically ill patients.^5^ Generally trauma triage protocols incorporate physiologic criteria, anatomic criteria, mechanism of injury and special considerations (age, comorbidities, etc).^7^ The 2011 Guidelines for Field Triage of Injured Patients from the Centers for Disease Control and Prevention (CDC) recommend a stepwise approach designed to identify serious injuries as early as possible during the prehospital assessment. Step one assesses vital signs and Glasgow Coma Scale (physiologic); step two assesses visible injuries such as penetrating injuries, crush injuries or long bone fractures (anatomic); step three assesses for high risk mechanisms including auto versus pedestrian or falls from a significant height (mechanism); and step four assesses for other complicating factors such as anticoagulated, pediatric, elderly, burned or pregnant patients among other unique situations (special considerations). As soon as one of these criteria is met, the EMS provider should make the decision to transport to a trauma center.^13^ These guidelines include core elements meant to be adapted to the needs of each individual EMS system, and thus protocols across the country differ slightly. Multiple studies have assessed the sensitivity and specificity of various triage protocols for determining if an injured patient needed transportation to a trauma center and have shown variability among different protocols.^6^,^8^–^12^ Additionally, there is variability in uptake of these guidelines across EMS systems following guideline revisions.^24^ The accuracies of each piece or the sum of these trauma triage protocols are relatively unknown.

Although the NEDS does not contain data regarding the mode of transportation to the ED we can estimate the rate of arrival by privately owned vehicle (POV) versus EMS from other studies. In one study using the National Trauma Data Bank, 12.6% of patients with gunshot wounds were transported to 182 trauma centers by POV.^25^ In a statewide study, 9.6% of all injured patients were transported to any trauma center by POV.^26^ In a regional study, 6.5% of patients with cervical spine injuries were transported to three trauma centers by POV.^27^ Although the mode of arrival of adult injured patients to non-trauma centers has not yet been assessed, these studies provide a starting point, estimating >85% of injured patients arrive to trauma centers via EMS. A high proportion of arrival via EMS reinforces the influence of pre-hospital trauma triage protocols on rates of under- and overtriage.

Our results highlight an opportunity to improve prehospital trauma triage protocols, particularly with prehospital provider perception of the severity of mechanism of injury, as undertriage was found to be associated with falls. EMS protocols and prehospital providers may be more likely to underestimate the severity of injury from falls relative to more visually obvious mechanisms of injury due to firearms and MVT. It has also been shown that older adults with falls who die soon after hospital arrival are often transported to non-trauma centers because the severity of their injuries is not recognized in the field.^28^ In addition to potential unrecognized injury secondary to the trauma from the fall, other potentially lethal medical causes of the fall such as syncope and associated high risk cardiac events, spontaneous intracranial hemorrhage or other severe metabolic derangements may be under-recognized causes of ED deaths. Recognition of these subtle presentations of severe trauma and potential serious medical causes of falls in older adults should be a target for improvement in prehospital trauma triage protocols.

Some geographic and socioeconomic differences in rates of undertriage were not anticipated. For example, people living in the Northeast have been shown to have the closest proximity to trauma centers,^18^,^29^ yet in our study were more likely to be undertriaged compared to the Midwest. This suggests that factors other than distance from a trauma center may account for this degree of undertriage. One variable that potentially affects prehospital adherence to trauma triage protocols is patient preference. For example, one study showed in approximately half of injured patients, EMS providers indicated patient preference as the reason for selecting the destination hospital.^30^ Perhaps this geographic difference in undertriage is a reflection of patient/family/EMS preference, lower utilization of EMS, or need for improvement of regionalized trauma systems in this area. Other factors such as hospital density, road conditions and ED divert status likely impact EMS triage decisions and would be an interesting direction for further study.

Trauma patient deaths with higher median household income were more likely to die in a non-trauma center than poorer patients. This finding was unanticipated given the general assumption that higher socioeconomic status leads to better access to medical care. One possible explanation could be that many trauma centers are located in inner cities and thus lower socioeconomic status populations, which are frequently in the same location, may have better access to these trauma centers. Further research is needed to determine potential reasons for this disparity.

We also found that female sex was associated with undertriage, consistent with a prior state-level study that reported female moderate to severely injured trauma patients at non-trauma centers were less likely to be properly transferred to a trauma center than men.^15^ While the cause of this disparity is unknown, we speculate that since women account for fewer trauma deaths than men overall, the severity of injury may be underestimated. Sex disparities have been noted in other areas of acute care including cardiac emergencies where men generally receive more aggressive early management than women.^31^

## LIMITATIONS

This study is a secondary analysis and like any similarly conducted study cannot determine causality. In addition, the database relies on administrative data, potentially leading to coding errors or missing data. Some data elements are not available in the NEDS, such as race/ethnicity or physiologic/anatomic prehospital triage criteria. Additionally, some EMS trauma triage protocols may dictate that if a patient has an emergent airway threat or other serious form of instability, he should be transported to the nearest hospital even if it is not a trauma center. This could account for an unknown portion of our patient sample categorized as undertriaged. Furthermore, we were unable to determine the precise reason for death, and whether the trauma/injury diagnosis was truly the cause of death. Though we assume that most gravely injured patients would be transported to the hospital by EMS, data are not available in the NEDS for mode of transport to the ED. Data were also not available for the geographic location of the injury or distance to closest hospital or trauma center, limiting our ability to fully address prehospital triage decisions. We also considered using the Injury Severity Score to study moderate and severely injured patients, but the use of this score is limited and can be inaccurate because often patients are too unstable to complete imaging needed to assess all injury categories prior to hospital transfer; the full extent of the ISS is not determined until the end of hospitalization and these data after transfer would not be available in the NEDS.

## CONCLUSION

High numbers of trauma patients who died in EDs were undertriaged to non-trauma centers, even in urban areas, where trauma centers are more accessible. Sex and other demographic disparities may impact trauma triage decisions in urban areas. These differences represent targeted opportunities to improve triage of specific populations to trauma centers.

## Figures and Tables

**Table 1:** Baseline characteristics of trauma-related emergency department (ED) visits in the 2010 National Emergency Department Sample.

	ED deaths (n=3,971)		Survived ED visits (n=4,975,715)	
	
Characteristics	n	Weighted % (95% CI)	n	Weighted % (95% CI)
Demographics				
Age, years				
18–34	1331	33.9 (32.4–35.5)	1,882,605	37.9 (37.8–37.9)
35–49	805	19.9 (18.7–21.3)	1,257,935	25.1 (25.1–25.1)
50–64	772	19.5 (18.2–20.9)	911,985	18.3 (18.3–18.4)
≥65	1063	26.6 (25.2–28.1)	937,768	18.7 (18.7–18.8)
Female sex	1126	28.1 (26.6–29.6)	2,512,067	50.1 (50.1–50.2)
Median household income				
$1–40,999	1177	30.1 (28.6–31.6)	1,474,500	29.7 (29.7–29.8)
$41,000–50,999	1072	27.1 (25.7–28.6)	1,345,638	27.1 (27.1–27.2)
$51,000–66,999	831	20.6 (19.3–22.0)	1,112,678	22.1 (22.0–22.1)
≥$67,000	686	17.1 (15.9–18.4)	941,503	18.8 (18.7–18.8)
Primary payer				
Medicare	901	22.6 (21.2–24.0)	1,069,394	21.4 (21.4–21.4)
Medicaid	341	8.7 (7.8–9.7)	716,103	14.4 (14.4–14.4)
Private	1086	28.4 (26.9–29.9)	1,634,268	33.4 (33.3–33.4)
Self-pay	1358	34.1 (32.5–35.6)	1,067,111	21.1 (21.1–21.1)
No charge/other	255	6.3 (5.6–7.1)	476,157	9.7 (9.7–9.8)
Weekend arrival	1316	33.1 (31.5–34.6)	1,506,155	30.4 (30.3–30.4)
Month of arrival				
January–March	571	18.4 (17.2–19.7)	934,814	22.1 (22.1–22.2)
April–June	739	22.0 (20.6–23.4)	1,120,229	26.5 (26.5–26.6)
July–September	877	22.3 (20.9–23.7)	1,165,350	27.7 (27.5–27.6)
October–December	900	22.4 (21.0–23.7)	1,008,723	23.8 (23.8–23.9)
Hospital characteristics				
Region				
Northeast	678	16.7 (15.6–17.8)	965,508	20.2 (20.2–20.2)
Midwest	784	22.0 (20.7–23.3)	1,092,194	24.6 (24.6–24.6)
South	1752	42.1 (40.6–43.6)	2,120,934	38.4 (38.3–38.4)
West	757	19.3 (18.0–20.5)	811,657	16.8 (16.7–16.8)
Trauma center	2021	55.5 (54.0–57.0)	1,532,579	35.0 (35.0–35.0)
Teaching/urban-rural status				
Metropolitan non-teaching	1437	32.5 (31.1–33.9)	2,365,176	43.4 (43.3–43.4)
Metropolitan teaching	1864	50.1 (48.5–51.6)	1,742,525	37.0 (37.0–37.0)
Micropolitan/rural (teaching and non-teaching)	670	17.5 (16.3–18.8)	882,592	19.6 (19.6–19.6)
Ownership				
Private	14222	21.3 (20.1–22.5)	1,536,808	26.4 (26.4–26.5)
Collapsed (public or private)	2735	73.9 (72.6–75.1)	3,059,866	67.4 (67.3–67.4)
Public- government, non-federal	3321	4.9 (4.3–5.5)	393,619	6.2 (6.2–6.2)
Emergency department (ED) volume				
<10,000	208	5.8 (5.0–6.8)	255,915	5.7 (5.7–5.7)
10,000–19,999	283	7.3 (6.4–8.2)	482,572	10.2 (10.1–10.2)
20,000–39,999	875	21.1 (19.9–22.5)	1,396,101	27.8 (27.8–27.9)
40,000–59,999	1059	28.4 (27.0–30.0)	1,141,757	23.0 (23.0–23.0)
60,000–79,999	631	14.8 (13.8–15.9)	804,751	15.3 (15.2–15.3)
>80,000	915	22.6 (21.3–23.9)	909,197	18.0 (18.0–18.1)
Safety net status	2705	67.5 (66.0–69.0)	3,189,345	62.3 (62.2–62.3)
Mechanism of injury				
Cutting or piercing	147	3.7 (3.1–4.3)	400,718	8.1 (8.1–8.1)
Drown	106	2.7 (2.2–3.3)	1,437	0.03 (0.03–0.03)
Fall	446	11.1 (10.1–12.1)	1,407,804	28.2 (28.2–28.2)
Fire	55	1.3 (1.0–1.8)	66,530	1.4 (1.3–1.4)
Firearm	745	19.0 (17.8–20.3)	13,518	0.3 (0.3–0.3)
Machinery	14	0.4 (0.2–0.7)	26,011	0.5 (0.5–0.5)
Motor vehicle trauma	1209	30.7 (29.2–32.2)	566,551	11.3 (11.3–11.4)
Natural/environmental	73	1.8 (1.4–2.2)	192,578	3.8 (3.8–3.9)
Poison	182	4.7 (4.0–5.6)	153,324	3.1 (3.0–3.1)
Struck by or against	78	2.0 (1.5–2.6)	575,861	11.6 (11.6–11.6)
Suffocation	186	4.5 (3.9–5.2)	7,356	0.2 (0.2–0.2)
Other	756	18.9 (17.7–20.2)	1,592,205	32.1 (32.1–32.1)

Note: n represents raw (unweighted) number of observations.

**Table 2:** Patient and hospital characteristics for trauma patients who died in non-trauma vs trauma hospitals among urban emergency departments (EDs).

Characteristics	Non-trauma hospital		Trauma hospital	

	n	% (95% CI)	n	% (95% CI)
Total	1,320	35.6 (34.1–37.1)	1,981	64.4 (62.9–65.9)
Demographics				
Age, years				
18–34	371	28.1 (25.7–30.6)	757	38.2 (36.0–40.4)
35–49	258	19.4 (17.3–21.6)	389	19.5 (17.7–21.4)
50–64	272	20.6 (18.4–22.9)	348	17.6 (15.9–19.4)
≥65	419	32.0 (29.5–34.6)	487	24.8 (22.9–26.9)
Female sex	424	32.7 (30.2–35.3)	520	25.9 (23.9–27.9)
Median household income				
$1–40,999	274	20.3 (18.2–22.6)	610	31.5 (29.4–33.7)
$41,000–50,999	349	25.9 (23.6–28.4)	495	25.0 (23.1–27.1)
$51,000–66,999	348	26.5 (24.1–29.0)	399	20.2 (18.3–22.1)
≥$67,000	294	23.2 (21.0–25.7)	350	17.0 (15.4–18.7)
Primary payer				
Medicare, private and other	704	54.1 (51.3–56.8)	923	47.4 (45.1–49.7)
Medicaid, self-pay and no charge	610	45.9 (43.2–48.7)	1,036	52.6 (50.3–54.9)
Weekend arrival	398	30.2 (27.8–32.8)	688	34.4 (32.2–36.7)
Month of arrival				
January–March	265	20.7 (18.6–23.0)	358	17.8 (16.2–19.7)
April–June	257	19.8 (17.7–22.1)	435	21.4 (19.6–23.4)
July–September	302	23.3 (21.1–25.7)	438	21.3 (19.5–23.2)
October–December	304	23.7 (21.4–26.1)	396	19.4 (17.7–21.2)
Hospital characteristics				
Region				
Northeast	223	20.0 (17.8–22.3)	395	17.2 (15.8–18.7)
Midwest	186	16.6 (14.6–18.8)	462	23.4 (21.8–25.2)
South	620	42.2 (39.7–44.8)	742	39.8 (37.8–41.9)
West	291	21.3 (19.2–23.5)	382	19.5 (18.0–21.2)
Mechanism of injury				
Motor vehicle trauma	310	23.1 (20.9–25.5)	709	35.6 (33.4–37.9)
Firearm	182	13.6 (11.9–15.6)	467	23.9 (22.0–25.9)
Fall	171	13.0 (11.3–14.9)	196	9.6 (8.4–11.1)
Other	657	50.3 (47.6–53.0)	612	30.8 (28.7–33.0)

**Table 3:** Multivariable logistic regression for characteristics of emergency department trauma deaths associated with triage to an urban trauma center vs. an urban non-trauma center.

Characteristics	Adjusted odds ratio	95% CI
Demographics		
Age, years		
18–34	Referent	-
35–49	0.86	0.69–1.07
50–64	0.74	0.59–0.92
≥65	0.78	0.62–1.00
Female sex	0.83	0.70–0.99
Median household income		
$1–40,999	Referent	-
$41,000–50,999	0.65	0.53–0.81
$51,000–66,999	0.52	0.42–0.65
≥$67,000	0.54	0.43–0.69
Primary payer		
Medicare, private and other	Referent	-
Medicaid, self-pay and no charge	0.97	0.81–1.17
Weekend arrival	1.13	0.97–1.33
Month of arrival		
January–March	Referent	-
April–June	1.23	0.97–1.33
July–September	1.02	0.81–1.29
October–December	0.89	0.70–1.13
Hospital characteristics		
Region		
Northeast	Referent	-
Midwest	1.88	1.47–2.40
South	0.80	0.65–0.99
West	1.17	0.93–1.48
Mechanism of injury		
Motor vehicle trauma	Referent	-
Firearm	0.90	0.71–1.14
Fall	0.50	0.38–0.66
Other	0.37	0.31–0.45
